# Editorial: Toxicological profiles and pharmacological properties of synthetic cannabinoids: From chemical and analytical issues to fatal and non-fatal intoxications

**DOI:** 10.3389/fpsyt.2023.1153522

**Published:** 2023-02-20

**Authors:** Arianna Giorgetti, Rossana Cecchi, Volker Auwärter

**Affiliations:** ^1^Unit of Legal Medicine, Department of Medical and Surgical Sciences, University of Bologna, Bologna, Italy; ^2^Department of Medicine and Surgery, Institute of Legal Medicine, University of Parma, Parma, Italy; ^3^Faculty of Medicine, Institute of Forensic Medicine, Forensic Toxicology, Medical Center, University of Freiburg, Freiburg im Breisgau, Germany

**Keywords:** new psychoactive substance (NPS), forensic toxicology, synthetic cannabinoid (SC), translational studies, drug use disorder

Since the discovery of the cannabinoid receptors in the central nervous system (CNS), that mediate the psychotropic effect of cannabis, a multitude of synthetic cannabinoid receptor agonists (SCRAs) has been developed to explore and probe the endocannabinoid system and to gather information on their potential therapeutic use. Prompted by the possibility of treating cancer pain and inflammatory diseases with cannabis, cannabinoid receptor ligands and in particular agonists started being synthetized as potential drug candidates. While in most cases not progressing to clinical trials, SCRAs have become a rapidly growing class of drugs of abuse, within the wider context of the new psychoactive substances (NPS) phenomenon. The first detection of SCRAs in herbal blends and “Spice” products dates back to 2008 (1) and, since then, they came into the focus of attention in the general public and particularly in the forensic science community. Although the number of new compounds per year monitored by the European Monitoring Centre for Drugs and Drug Addiction (EMCDDA) slightly decreased since 2014-2015 (2), SCRAs still represent a serious public health and social issue.

The dynamic market, subjected to rapid changes on national as well as international level, and the variety in the molecular structures with far more than 200 individual molecules being monitored by the EMCDDA today, represent challenges for the control of these compounds as well as for the development of analytical strategies aimed at detecting SCRAs in seized material or biological fluids. Previous research has shown that one of the reasons to abuse SCRAs instead of “classical” drugs of abuse might be the motivation to escape detection in routine drug tests based on immunoassays. However, different target populations might consume SCRAs for distinctive reasons, e.g., ease of availability and easy smuggling likely motivate the use of NPS in prisons and in other custodial settings. Due to online selling, SCRAs can easily achieve very young people and it is foreseeable that in the future even more young users will get in touch with these substances. This represents a health risk for the new generations, whose chances to achieve the expected goals might decrease, given the impairment to a still immature brain. Furthermore, it has not to be underestimated the risk of permanent disabilities or even death due to the effects of NPS in low dosage respect to natural cannabinoids.

Even though prevalence data and knowledge on characteristics of abuse might allow the development of specific prevention strategies, in many countries the prevalence is unknown or hard to estimate due to analytical limitations, including the degradation of these substances when defrosted several times. The absence of data might lead to an under-estimation of the phenomenon, which is a further barrier for the development of prevention strategies and countermeasures.

Additional challenges posed by SCRAs to the field of forensic toxicology are represented by the relatively short detection window in blood as well as by the lack of pharmacokinetic data, especially on the metabolism of new compounds, which are needed in order to develop targeted methods for the analysis of urine, the most common matrix for abstinence testing. In the context of acute fatal or non-fatal intoxications, understanding the role of NPS and SCRAs is hampered by the absence of data regarding their pharmaco-toxicological profiles. Experimental data on affinity and potency of SCRAs to the cannabinoid receptors might be generated *in vitro* or *in silico*, allowing to assist the assessment of their toxicity, their capability to induce impairment of CNS functions and of the risk of lethal outcome. Animal models can provide useful experimental data on SCRA effects, without the ethical restrictions usually applied to research on humans, but are affected by issues regarding comparability of doses and toxicity in humans. Human data are usually connected only to case reports or small case series, and are further complicated by factors like tolerance and interindividual pharmacokinetic/genetic variability, which hampers the possibility to clearly define therapeutic, toxic or fatal concentration ranges.

Some of these issues are addressed by the studies collected under this Research Topic. Sparkes et al. tackled the appearance of SCRAs bearing novel tail substituents by detailing synthesis, characterization, and pharmacological evaluation of recently detected compounds applying *in silico* and *in vitro* studies. Alías-Ferri et al. analyzed the prevalence of SCRA use among patients with opioid use disorder (OUD), suggesting that in this specific setting they might be used as substitutes for cannabis to avoid detection in clinical tests. Barbieri et al. investigated the effects of acute administration of a naphthoylindole compound on sensory-motor and motor responses, memory retention and on hippocampal/cortical functionality in an animal model. Lastly, Orazietti et al. provided a systematic literature review of animal and human data, highlighting the effects of SCRAs on psychomotor functions and the risks for road safety.

Overall, this issue represents a further confirmation that, in order to increase the knowledge regarding SCRAs and to answer the multiplicity of emerging questions, a broad spectrum of methodologies has to be applied, including *in vitro* and *in vivo* experimental studies as well as epidemiological and analytical research. From preclinical bioassays and animal models to human effects, the collection herein reported also highlights that the coverage of SCRAs in clinical and forensic science requires a multidisciplinary approach, not only when evaluating forensic cases but also in basic research. Future studies aiming at combining the evidence arising from the various research settings is encouraged. We hope that this collection will contribute to the great progress this field has seen in recent years.

## Author contributions

AG wrote the first draft. RC and VA revised and supervised the work. All authors listed have made a substantial, direct, and intellectual contribution to the work and approved it for publication.
